# Co-delivery of Cisplatin(IV) and Capecitabine as an Effective and Non-toxic Cancer Treatment

**DOI:** 10.3389/fphar.2019.00110

**Published:** 2019-02-19

**Authors:** Xiao Xiao, Ting Wang, Leijiao Li, Zhongli Zhu, Weina Zhang, Guihua Cui, Wenliang Li

**Affiliations:** ^1^School of Pharmacy, Jilin Medical University, Jilin, China; ^2^Center for Biomaterials, Jilin Medical University, Jilin, China; ^3^Department of the Gastrointestinal Surgery, The First Hospital of Jilin University, Changchun, China; ^4^Jilin Provincial Science and Technology Innovation Center of Optical Materials and Chemistry, School of Chemistry and Environmental Engineering, Changchun University of Science and Technology, Changchun, China; ^5^Common Subjects Department, Shangqiu Medical College, Henan, China

**Keywords:** co-delivery, cisplatin(IV), capecitabine, cancer treatment, composite micelles

## Abstract

A strategy for preparing composite micelles (CM) containing both cisplatin(IV) [CisPt(IV)] prodrug and capecitabine using a co-assembly method is described in this study. The CM are capable of an effective release of the anticancer drug cisplatin(II) [CisPt(II)] and capecitabine via acid hydrolysis once they are internalized by cancer cells. Moreover, the CM display a synergistic effect *in vitro* and the combination therapy in the micellar dosage form leads to reduced systemic toxicity and enhanced antitumor efficacy *in vivo*.

## Introduction

Utilization of single small molecule anticancer agents in clinical thermotherapy is very rare owing to the rapid development of drug resistance in tumor cells (Di Francesco et al., 2002; Ahmad, 2010). Hence, drug combination is predominantly used in the clinical setting (Joensuu et al., 1998; Feldmann et al., 2007; Woodcock et al., 2011). Combination therapy presents its own set of advantages, related to the improved medication compliance and the enhanced ability to formulate combined drug profiles. From the pharmacokinetic stand point, the positive effects and adverse effects of a combination therapy may be specific to the relative dosages providing a simpler overview compared to single drug profiles. Moreover, the interaction between the individual drugs in a specific combination drug may also provide additional positive effects which may be absent in the individual drug profiles. The active ingredients used in combination drugs rarely exhibit any adverse interactions and are scrupulously reviewed by the Food and Drug Administration (FDA) in the United States (Love et al., 1989; Sitzia and Huggins, 1998). Among the combination chemotherapy regimens, co-administration of capecitabine and platinum-based drugs is the most common.

Platinum-based anticancer agents are one of the most commonly used chemotherapeutic drugs in the treatment of various solid tumors (Lebwohl and Canetta, 1998; Pasettoa et al., 2006; Xiao et al., 2017; Shen et al., 2018). However, almost all platinum drugs possess some inherent and serious side effects which influence drug resistance (Sharma et al., 2005; Windebank and Grisold, 2008). Cisplatin (cis-diamminedichloroplatinum, CDDP) is the first approved platinum drug, which has been used as a standard chemotherapeutic agent for more than 30 years (Weiss and Christian, 1993; Rosenberg and Lippert, 1999). Due to the low concentration of chloride, cisplatin is hydrolyzed inside the cell and is converted to the highly reactive species [Pt(NH_3_)_2_Cl(OH_2_)]^+^, which creates 1,2-GpG intrastrand adducts within the DNA. The adducts inhibit DNA transcription and replication, ultimately leading to cancer cell apoptosis (Jamieson and Lippard, 1999; Boulikas et al., 2007). Nevertheless, severe side effects, including nephrotoxicity, neurotoxicity, ototoxicity and myelosuppression, limit the clinical application of cisplatin. Moreover, the intrinsic and acquired resistance developed by various cancers is another main reason for limiting application of cisplatin (Madias and Harrington, 1978; Ekborn et al., 2000).

Fluorouracil (5-FU) is one of the most widely used medications for the treatment of various cancers, such as colon, esophageal, stomach, pancreatic, breast, and cervical cancers. Unfortunately, 5-FU is distributed to both the healthy and the cancer cells following systemic administration making the drug cytotoxic and leading to death of healthy cells (Arkenau et al., 2003). Capecitabine is an antitumor fluoropyrimidine carbamate, which is very specific only toward cancer cells owing to the activation of tumor-specific enzymes (Ssif et al., 2008).

In comparison to single drug anti-cancer treatment, enhanced tumor therapy efficiency is often observed in patients administered a combination therapy (Joensuu et al., 1998; Feldmann et al., 2007; Woodcock et al., 2011). For example, Xeloda is indicated for the first-line treatment of advanced gastric cancer and is a combination of capecitabine and platinum regimen (DÍaz-Rubio et al., 2002). However, ensuring a correct dosage of a given combination drug in the context of endocytosis within the cell remains a huge challenge in small molecule-based combination therapy. In addition, unfavorable side effects likely accompany the combination of small molecule drugs. To improve the therapeutic efficacy and to reduce the side effects, nanocarriers are used to encapsulate the combination drugs (Duncan, 2003; Kwangjae et al., 2008; Maeda, 2010; Bae and Park, 2011; Tao et al., 2013, 2014, 2016, 2017, 2018; Zhu et al., 2018; Ling et al., 2019). Nanomedicine approach can assure selective accumulation of the drug at tumor sites via the enhanced permeation and retention effect, in turn protecting the drug from premature degradation and blood clearance.

**SCHEME 1:**
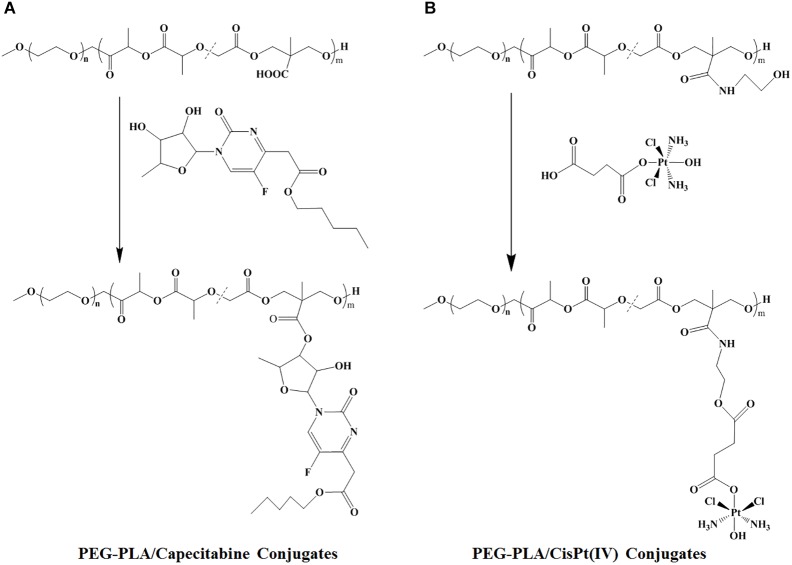
Synthesis of **(A)** PEG-PLA/capecitabine conjugates and **(B)** PEG-PLA/CisPt(IV) conjugates.

**SCHEME 2:**
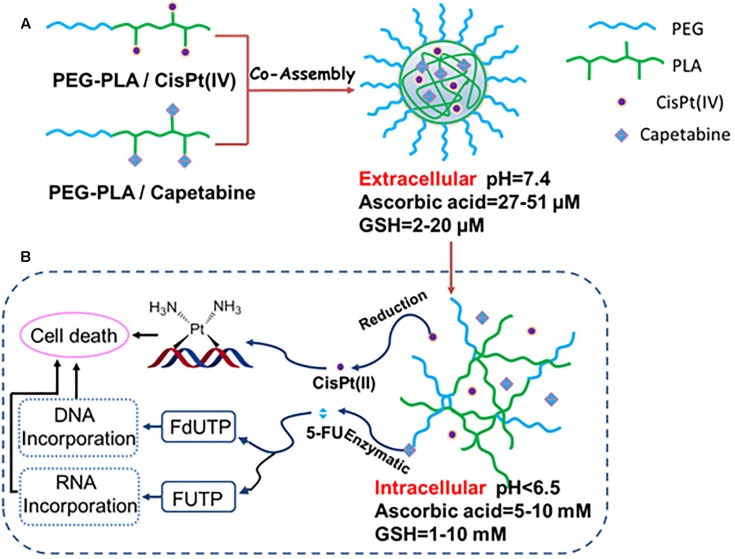
**(A)** Preparation of drug combination micelles and **(B)** a schematic representation of hypothetical cellular pathways and mechanism of action of drug combination micelles.

In this work, two polymer-drug conjugates [MPEG-*b*-P(LA-*co*-MCC)-COOH/capecitabine and MPEG-*b*-P(LA-*co*-MCC)-OH/CisPt(IV)] were synthesized (Scheme 1). Because of the amphiphilic properties of these polymer-drug conjugates, they were able to be co-assembled into composite micelles (CM) creating a combination therapy drug delivery system (Scheme 2).

The resulting polyprodrug-based combination chemotherapy regimen displays many unique features. Firstly, the relative drug doses of cisplatin(IV) and capecitabine can be easily adjusted by modulating the ratio of PEG-PLA/CisPt(IV). Next, the polymer micelles-based prodrug delivery system provides the active drugs [cisplatin(II) and 5-FU] with double protection. Specifically, both cisplatin(IV) and capecitabine are located in the core of the CM resulting in effective protection mechanism. Additionally, the other protection stems from the prodrug formulation. After a series biotransformation events, cisplatin(IV) and capecitabine get converted to active agents’ cisplatin(II) and 5-FU, respectively. Therefore, it is critical for small molecule drug delivery to assure constant levels of the two drugs in the circulation. Importantly, the polymer micelle-based prodrug strategy provides an effective protection for the drugs. Compared to other small molecule-based combination therapies, our method displays significantly lower systemic toxicities. Finally, once the polymeric CM enter the tumor cells, prodrugs cisplatin(IV) and capecitabine will be released by the hydrolysis of PEG-PLA/cisplatin(IV) and PEG-PLA/capecitabine conjugates in the micelles as shown in Scheme 2. Moreover, cisplatin(IV) can be converted to cisplatin(II) under the intracellular reducing conditions (i.e., high concentration of glutathione (GSH) and ascorbic acid). Cisplatin(II) can also become conjugated to basic groups of DNA, in turn killing the tumor cells. Additionally, capecitabine can be converted to 5-FU under the enzymatic catalysis. Specifically, 5-FU can be converted to fluorouridine triphosphate (FUTP) and fluorodeoxyuridine triphosphate (FdUTP), which are then incorporated into RNA and DNA, respectively, ultimately leading to cell death.

## Materials and Methods

### Materials

The polymers MPEG-*b*-P(LA-*co*-MCC)-COOH and MPEG-*b*-P(LA-*co*-MCC)-OH were synthesized as previously described. *N*,*N*’-dicyclohexylcarbodiimide (DCC) and *N*-hydroxybenzotriazole (HOBt) were purchased from Sigma-Aldrich. CisPt(IV) prodrug was synthesized as reported in our earlier work and capecitabine was purchased from the Zhejiang Haizheng Pharmacy Co. Ltd. All other chemicals and solvents were used without additional purification steps.

### General Measurements

^1^H-NMR spectra were measured by a Unity-300 MHz NMR spectrometer (Bruker) at room temperature. Fourier Transform infrared (FT-IR) spectroscopy was performed using a Bruker Vertex 70 spectrometer. A Quattro Premier XE system (Waters) equipped with an electrospray interface was used for the mass spectroscopy (ESI-MS) assessments. The total platinum (Pt) content in the polymer-Pt(IV) conjugates and in samples obtained from the dialysis bags in drug release experiments was determined by inductively coupled plasma optical emission spectrometer (ICP-OES, iCAP 6300, ThermoScientific, United States). The quantitative determination of trace levels of Pt was measured by ICP-MS (Xseries II, ThermoScientific, United States). Size and size distribution of micelles were determined by dynamic light scattering (DLS) with a vertically polarized He–Ne laser (DAWN EOS, Wyatt Technology, United States). The morphology of the polymer micelles was evaluated by transmission electron microscope (TEM) performed on a JEOL JEM-1011 electron microscope. Particle size and zeta potential measurements were conducted on a Malvern Zetasizer Nano ZS. (Zetasizer Nano ZS is a high performance dual angle particle size and molecular size analyzer that uses DLS combined with “NIBS” optics to enhance the detection of aggregates, as well as small or diluted samples, and poles. Low or high concentration sample).

### Synthesis of N-Hydroxy-Succinamide (NHS) Ester of MPEG-*b*-P(LA-*co*-MCC)

MPEG-*b*-P(LA-*co*-MCC) (0.5 g, 0.0625 mmol) was dissolved in 5 mL of CH_2_Cl_2_ in a flask, to which DCC (128.9 mg, 0.625 mmol), NHS (71.9 mg, 0.625 mmol), and DMAP (76.35 mg, 0.625 mmol) were added. The reaction mixture was constantly stirred in an ice bath for 24 h. Next, it was filtered to remove 1,3-dicyclohexylurea (DCU). The filtrate was then added dropwise to cold diethyl ether (50 mL), the resultant precipitate was collected by filtration and dried under vacuum to obtain the NHS ester of MPEG-*b*-P(LA-*co*-MCC).

### Synthesis of MPEG-*b*-P(LA-*co*-MCC)-OH

The NHS ester of MPEG-*b*-P(LA-*co*-MCC) (0.5 g, 0.0625 mmol) was dissolved in dried CH_2_Cl_2_ and 2-aminoethyl alcohol (4.6 mg, 0.075 mmol) was added to the polymer solution. The reaction mixture was stirred at room temperature for 24 h and then precipitated with ether to obtain the final product MPEG-*b*-P(LA-*co*-MCC)-OH.

### Synthesis of MPEG-*b*-P(LA-*co*-MCC)-OH/Pt(IV) Conjugates

Cisplatin(IV)-COOH [abbreviated as CisPt(IV)] was synthesized as described previously. CisPt(IV) was conjugated to the polymer MPEG-*b*-P(LA-*co*-MCC)-OH with pendant hydroxyl groups using N-dicyclohexyl carbodiimide/4-dimethylaminopyridine (DCC/DMAP). Briefly, CisPt(IV) (43.3 mg, 0.081 mmol) was first dissolved in dried N,N-Dimethylformamide (DMF) (1 mL) in a flask under stirring conditions. The polymer MPEG-*b*-P(LA-*co*-MCC)-OH (0.5 g, 0.0625 mmol) was then dissolved in 3 mL of C_2_H_2_Cl_2_ and then added to the CisPt(IV) solution. Next, 128.9 mg of DCC, 76.35 mg of DMAP and 95.7 mg of HOBt were added into the solution. The reaction mixture was stirred for 2 days at room temperature. Afterward, the mixture was filtered to remove DCU. The filtrate was slowly added to diethyl ether to precipitate the crude product, which was then dried to yield a pale yellow powder. Next, the powder was dissolved in DMF and placed into a dialysis bag and dialyzed against water to remove unreacted CisPt(IV). Finally, the solution was lyophilized to obtain the MPEG-*b*-P(LA-*co*-MCC)-OH/Pt(IV) conjugates.

### Synthesis of MPEG-*b*-P(LA-*co*-MCC)/Capecitabine Conjugates

Capecitabine was conjugated to the polymer MPEG-*b*-P(LA-*co*-MCC) with pendant carboxyl groups using the DCC/DMAP/HOBt method. Briefly, capecitabine (43.3 mg, 0.081 mmol) was first dissolved in dried DMSO in a flask under stirring conditions and 0.5 g MPEG-*b*-P(LA-*co*-MCC) dissolved in 3 mL of C_2_H_2_Cl_2_ was then added. Afterward, 128.9 mg of DCC, 76.35 mg of DMAP, and 95.7 mg of HOBt were added. The reaction mixture was stirred for 2 days at room temperature. Next, it was filtered to remove DCU, precipitated by ether and collected by filtration. The product was dried to obtain a red powder. The powder was then dissolved in DMF and it was placed into a dialysis bag and dialyzed against water to remove unreacted capecitabine. Finally, following the dialysis, the solution was lyophilized to yield the MPEG-*b*-P(LA-*co*-MCC)/capecitabine conjugates.

### Preparation of MPEG-*b*-P(LA-*co*-MCC)-OH/Pt(IV) and MPEG-*b*-P(LA-*co*-MCC)/Capecitabine CM

The CM were prepared by nano-precipitation method. In brief, 50 mg of MPEG-*b*-P(LA-*co*-MCC)-OH/Pt(IV) conjugates and 50 mg of MPEG-*b*-P(LA-*co*-MCC)/capecitabine conjugates were dissolved in a flask filled with 10 mL of DMF. Next, the solution was added in a dropwise fashion into the flask under stirring to form a micellar solution. The solution was then dialyzed against water to remove DMF and then freeze-dried to obtain the MPEG-*b*-P(LA-*co*-MCC)-OH/Pt(IV) and MPEG-*b*-P(LA-*co*-MCC)/capecitabine CM.

### *In vitro* Drug Release of CM

The CM (5 mg) were dissolved in PBS (10 mL, 0.1 M, pH 7.4, or pH 5.5) and then placed into a pre-swelled dialysis bag (3500 MWCO), which was immersed into PBS (100 mL) at 37°C in a shaking culture incubator (Wang et al., 2012). At 1 h intervals, 1.5 mL of sample solution was withdrawn from the dialysate and measured for the Pt and capecitabine concentration using ICP-OES and HPLC, respectively. After sampling, fresh PBS (1.5 mL) was immediately added back into the incubation medium. The process was repeated for 8 h. The Pt released from the micelles was expressed as the percentage of the cumulative Pt or capecitabine outside the dialysis bag to the total Pt or capecitabine inside the micelles.

### Cell Lines and Cell Culture Conditions

Human Colorectal Tumor Cells HCT-8 (ATCC) were cultured in RPMI 1640 media (Hyclone, Logan, UT, United States) supplemented with 10% fetal bovine serum (FBS, Life Technologies, United States), 100 U/mL penicillin and 100 μg/mL streptomycin (Ameresco, Life Technologies, United States) at 37°C with 5% CO_2_.

### Cell Viability Studies

The HCT-8 cells harvested in a logarithmic growth phase were seeded in 96-well plates at a density of 1 × 10^4^ cells/well and incubated in 100 μL/well RPMI 1640 medium for 12 h. The medium was then replaced by CM with a Pt concentration ranging from 0.1 to 432 μM. To assess cell viability, 20 μL of MTT solution (5 mg/mL) was added and the plates were incubated for another 4 h at 37°C. Next, the culture medium containing MTT was removed and 150 μL of DMSO was added to dissolve the formazan crystals that were formed. Finally, the plates were shaken for 10 min and the absorbance was measured at 492 nm using a microplate reader.

### Synthesis of MPEG-*b*-P(LA-*co*-MCC)-OH/CisPt(IV) Conjugates

With CisPt(IV) in hand, we hoped to create a carrier polymer with hydroxyl groups. Therefore, MPEG-*b*-P(LA-*co*-MCC)-OH was synthesized according to our previous report (Xiao et al., 2012). CisPt(IV) was conjugated to the polymer MPEG-*b*-P(LA-*co*-MCC)-OH using DCC/DMAP method and the ICP-OES measurement of MPEG-*b*-P(LA-*co*-MCC)-OH/CisPt(IV) conjugates indicated a Pt content of 9.3 w/w%.

### Synthesis and Characterization of MPEG-*b*-P(LA-*co*-MCC)/Capecitabine Conjugates

**FIGURE 1:**
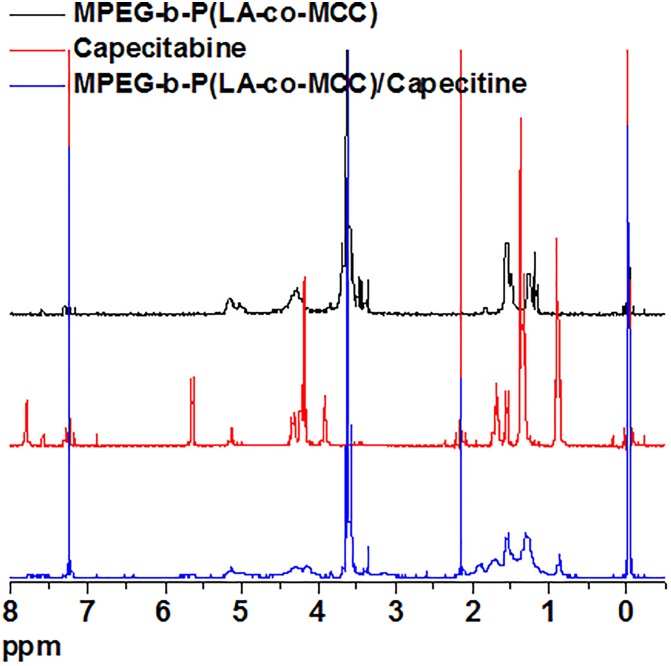
The ^1^H-NMR spectra of MPEG-*b*-P(LA-*co*-MCC) (black curve), capecitabine (red curve), and MPEG-*b*-P(LA-*co*-MCC)/capecitabine (blue curve).

**FIGURE 2:**
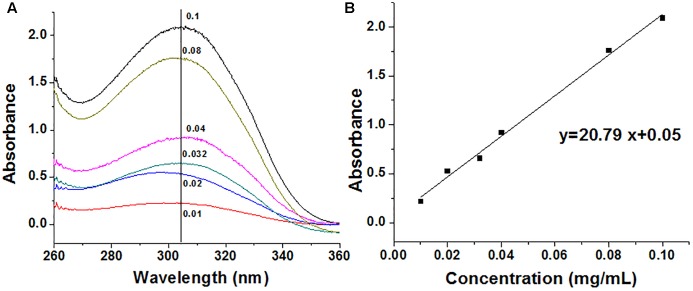
Determination of the capecitabine content in drug combination particles. **(A)** UV–vis spectra of stock solutions of capecitabine in water (mg/mL) and **(B)** standard curve of capecitabine stock solutions.

Capecitabine was conjugated to the polymer MPEG-*b*-P(LA-*co*-MCC) with pendant carboxyl groups using the DCC/DMAP/HOBt method. The ^1^H-NMR spectra of capecitabine, MPEG-*b*-P(LA-*co*-MCC) and MPEG-*b*-P(LA-*co*-MCC)/capecitabine in deuterated chloroform (CDCl_3_) solvent were collected and are summarized in Figure 1. Compared to the ^1^H-NMR spectrum of MPEG-*b*-P(LA-*co*-MCC), we identified additional new peaks in the MPEG-*b*-P(LA-*co*-MCC)/capecitabine conjugates. These peaks may likely be attributed to capecitabine. Moreover, we collected the UV–vis spectra of capecitabine solutions at different concentrations (0.01–0.1 mg/mL) and summarized the data in Figure 2A. Using the spectra, we were able to calculate the standard curve of absorbance at 304 nm vs. capecitabine concentration (Figure 2B). This standard curve and the UV–vis spectra of MPEG-*b*-P(LA-*co*-MCC)/capecitabine conjugates revealed that the capecitabine content in the conjugates was 17% w/w.

### Preparation of the Mixed Polymer-Drug Micelles

**FIGURE 3:**
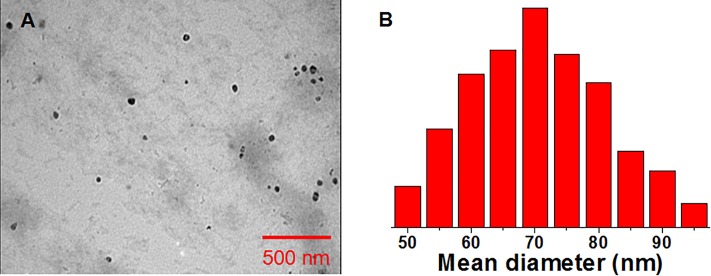
**(A)** TEM and **(B)** DLS characterization of the drug combination particles.

Drug combination particles were prepared by the self-assembly method. The obtained particles were spherical micelles with a mean diameter of 55 nm (according to TEM) and 110 nm (according to DLS) (Figure 3). The resulting particle size using the DLS was larger compared to the TEM assessment, likely due to the shrinkage during the TEM sample preparation process. The particles displayed a zeta potential of -4.4 mV, which is almost neutral, hence we expected the particles to have a good stability *in vivo*.

## Results and Discussion

### Drug Release

**FIGURE 4:**
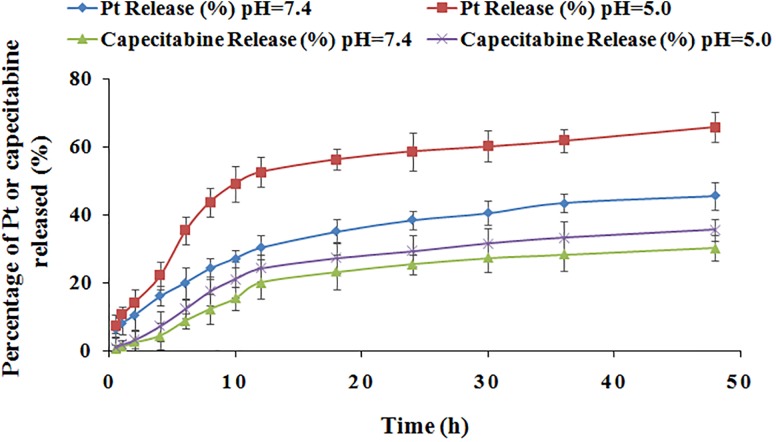
Pt and capecitabine release profiles in buffer solutions at pH 5.0 and pH 7.4.

Drug release experiments were performed at different pH values as stated in the literature (Choi et al., 1998). Data presented in Figure 4 show the release kinetics (0–50 h) for Pt and capecitabine in the combination drugs in two conditions at pH 5.0 and pH 7.4. Overall, the drug release curves showed that the two drugs displayed sustained release at both pH 5.0 and pH 7.0 and the maximum release occurred at approximately 12 h and then remained stable for 50 h. Moreover, the cumulative release percentages of Pt and capecitabine at pH 5.0 were higher compared to values at pH 7.4. Additionally, we observed that the Pt release was more sensitive to pH compared to the capecitabine release. Importantly, the intracellular compartments for the endosomes and lysosomes are acidic in cancer cells, allowing for the release of these two drugs into cancer cells and thereby improving the therapeutic effect.

### *In vitro* Cytotoxicity

**FIGURE 5:**
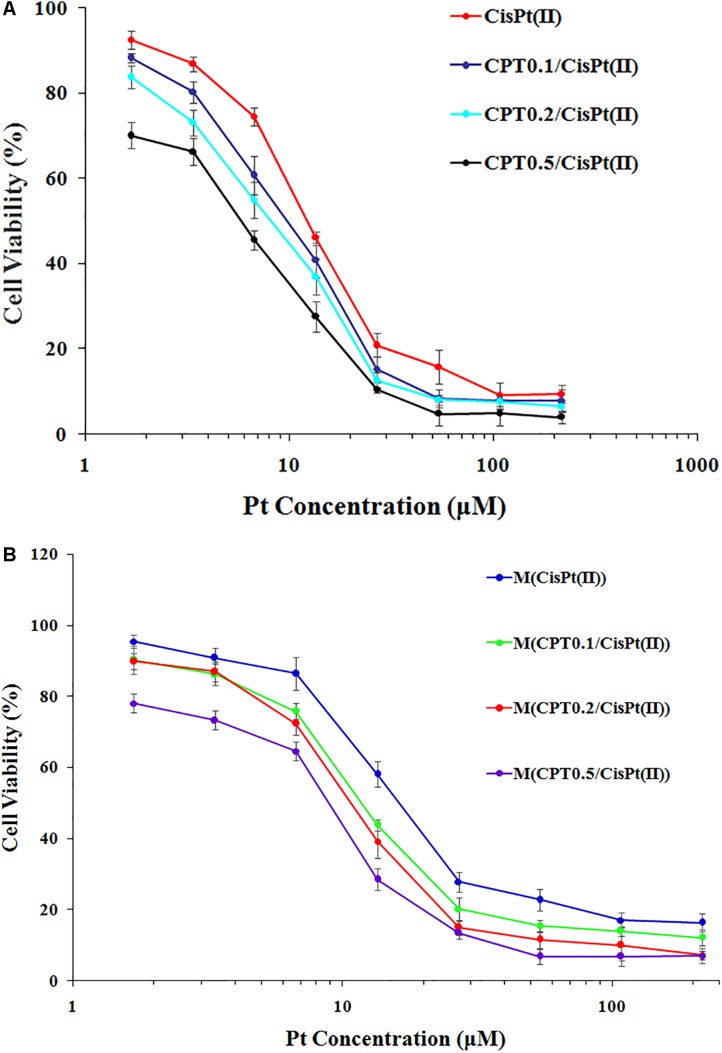
*In vitro* cytotoxicity profiles of **(A)** free CTP/CisPt(II) and **(B)** M[CPT/CisPt(II)] with various CPT/Pt ratios performed in HCT-8 cells at 48 h.

We calculated the IC_50_ values of the combination therapy of free drug capecitabine(CPT)/CisPt(II) combinations and mixed polymer-drug micelles M(CPT/CisPt(II)) with various CPT/CisPt(II) ratios in HCT-8 cells at 48 h (Table 1). We observed that both free drugs CPT/CisPt and mixed polymer-drug micelles M[CPT/CisPt(II)] exhibited an enhancement of combination potency accompanied by dose-response profiles being shifted toward lower drug concentrations when the CPT/Pt molar ratio increased (Figure 5).

**Table 1:** The IC50 values of free drug CPT/CisPt(II) combinations and mixed polymer-drug micelles M[CPT/CisPt(II)] with various CPT/CisPt(II) ratios evaluated in HCT-8 cells at 48 h.

Free drug combinations	IC_50_ (μmol/L)	Polymer-drug Micelles	IC_50_ (μmol/L)
CisPt(II)	13.66	M[CisPt(II)]	21.47
CPT_0.1_/Pt	9.62	M(CPT_0.1_/Pt)	13.82
CPT_0.2_/Pt	7.74	M(CPT_0.2_/Pt)	11.55
CPT_0.5_/Pt	5.03	M(CPT_0.5_/Pt)	7.40


At 48 h, the IC_50_ values of free CisPt(II) alone and M[CisPt(II)] were 13.66 and 21.47 μM, respectively. The IC_50_ values of both free drug combinations and the polymer-drug micelles were sharply reduced when the CPT/Pt molar ratio increased (Table 1). These data suggest that synergistic cytotoxicity of CPT/CisPt(II) formulations against HCT-8 cells was evident. More importantly, the mixed polymer-drug micelles, M[CPT/CisPt(II)], had similar synergistic effects in HCT-8 cells. In short, M(PTX/Pt) (PTX is short for paclitaxel) displayed significant time- and dose-dependent inhibitory effects allowing for the dissociation and the release of the drugs into the cells.

## Conclusion

In this study, we successfully created a synthetic strategy for drug co-delivery by conjugating and co-assembling, wherein two different drugs, including CisPt(II) based drugs, are enveloped into one carrier polymer allowing for the delivery of the combination therapy. Our method exhibited effective synergistic effect *in vitro* between the free CTP/CisPt(II) and the composite M(PTX/Pt) micelles. Furthermore, the polymer-based combination of capecitabine and cisplatin(IV) prodrug displayed safer and more efficacious inhibition of HCT-8 cell growth compared to the small molecules individually. We hope that our combination strategy can be extended to other anticancer drugs. Given the significant effect of the combination therapy, we believe this strategy will likely be utilized in the clinic in the foreseeable future.

## Author Contributions

LL, GC, and WL designed the experiments. XX and TW carried out the experiments and wrote the manuscript. ZZ and WZ helped analyzing the experimental results.

## Conflict of Interest Statement

The authors declare that the research was conducted in the absence of any commercial or financial relationships that could be construed as a potential conflict of interest.
